# Navigating intensive altered states of consciousness: How can the set and setting key parameters promote the science of human birth?

**DOI:** 10.3389/fpsyt.2023.1072047

**Published:** 2023-02-09

**Authors:** Orli Dahan

**Affiliations:** Department of Multidisciplinary Studies, Faculty of Social Sciences and Humanities, Tel-Hai College, Tel-Hai, Israel

**Keywords:** altered states of consciousness (ASC), birth experience, birth medicalization, human birth, intensive inner dynamic, life-altering experiences, sensitive feedback loop, set and setting

## Abstract

The subjective childbirth experience is crucial from a public health standpoint. There is a correlation between a negative childbirth experience and a poor mental state after birth, with effects that go far beyond the postpartum (PP) period. This paper offers a new approach as to how birthing experiences, and birth in general, can be navigated. The theory of set and setting proves that psychedelic experiences are shaped, first and foremost, by the mindset of an individual entering a psychedelic experience (set) and by the surroundings in which the experience happens (setting). In research on altered states of consciousness during psychedelic experiences, this theory explains how the same substance can lead to a positive and life-changing experience or to a traumatic and frightening experience. Because recent studies suggest that birthing women enter an altered state of consciousness during physiological birth (“birthing consciousness”), I suggest analyzing the typical modern birthing experience in terms of set and setting theory. I argue that the set and setting key parameters can help design, navigate, and explain many psychological and physiological elements of the human birth process. Thus, an operative conclusion that emerges from the theoretical analysis presented in this paper is that framing and characterizing the birth environment and birth preparations in terms of set and setting is a central tool that could be used to promote physiological births as well as subjective positive birthing experiences, which is currently a primary, yet unreached goal, in modern obstetrics and public health.

## 1. Introduction

This theoretical paper aims to introduce the critical parameters of “set and setting” from the field of psychedelic research to the field of childbirth research and argue that, if taken seriously, these parameters would significantly advance scientific understanding concerning the birth process and the factors that contribute to or hinder the physiological birth process as well as concerning polar birth experiences.

In spite of the many advances in the field of modern obstetrics, childbirth has become more dangerous to women in the Western world—because of the increase in highly medicalized birth (1, 2). Practices that were originally lifesaving became standard practices. However, these routine procedures do not seem to relate to a reduction in mortality ratios (3). The dangers of highly medicalized birth are both physiological and mental (4). Physiological recovery after natural and less medicated birth is generally far faster and easier than after a highly medicated birth, for example in terms of pelvic floor health (5–7), and according to many studies, the subjective experience of the birthing woman during childbirth affects her postpartum (PP) mental health (8). Acute stress and fear during childbirth hinder the birth process, necessitating medical interventions that have negative physiological and mental health consequences, compared to the physiological birth process with minimum medical intervention (9–11).^1^

Thus it is crucial to understand the factors that shape birth experiences. I propose that a fruitful way to do so would be through comparing physiological birth experiences to psychedelic experiences. There are at least four clear resemblances between the two phenomena.

First, both experiences are, in many cases, associated with altered states of consciousness: the phenomenology of both states is similar, and there is a probability that both have the similar brain mechanism of hypofrontality [see (12–14)]. Altered states of consciousness are typically experienced during activities such as meditation, hypnosis, daydreaming, dreaming, certain drug states, and prolonged running or other extreme sports activities. Dancing, swimming, hiking, swaying in prayer, fasting, or pain stimulations–may also lead to altered states of consciousness experiences in varying degrees (15).

Second, both birth experiences and psychedelic experiences, have an intensive inner dynamic (16, 17) derived from being an altered state of consciousness. This means that there are compound and dynamic emotional and physiological instabilities during childbirth (16). Not all altered states of consciousness always have such an intensive dynamic, for example, a meditative state.

Third, seemingly connected to this intensity, both can be analyzed in terms of surprising causal feedback loops. Causal feedback loops encompass response dynamic: the results of a certain variation, even a minor one, may strengthen, or contradict the initial variation. They introduce complex dynamics by which a shift in one feature may impact a different feature, which then is capable of impact the initial feature. Feedback loops can amplify an event in either a positive or negative direction (18). The birthing experience is created by an highly sensitive feedback loop, because the experience is produced and influences by many factors, that interact and sometimes interrupt one another, such as the mental states of the birthing woman before and during the event of birth, and the communication and interactions with other people in the birth environment (10, 16, 18, 19). Hallucinogens also may lead to these feedback loops (20). In fact, the significant impacts of psychedelics are the result of disruption in the processing of information in brain areas, particularly in the striato–thalamo–cortical feedback loops, with information coming from both internal and external stimuli (21, 22).

Fourth, there are two opposing, extreme subjective experiences possible for both phenomena. Psychedelic substances can generate a tremendously good experience–a “good trip”–but the same substance can sometimes generate the opposite sensation–a “bad trip” (23). A good psychedelic experience may have a persistent positive effect [see (24)]. Similarly, the physiological birth experience sometimes generates a feeling of joy that can be life-altering (e.g., enhancing self-esteem, boosting energy) (10, 11), but it sometimes can be felt as devastating and traumatic (8), even generating post-traumatic stress disorder (PTSD) (10). This effect is unique to birthing and psychedelic experiences. It seems that it is not so dramatically polarized in other altered states of consciousness, such as meditation, hypnosis, or a runner’s high (12). For example, I do not believe it is common to experience a traumatic marathon or meditation session. Perhaps this is because these activities can be stopped at will, while normally one cannot stop birthing at will once it is started or stop a psychedelic effect after a mind-altering substance has been taken.

On the face of it, this looks like an enigma: how can the same substance in the case of psychedelics, or the same physiological process in the case of childbirth, produce such extreme and opposing experiences, with such extreme and opposing consequences? For psychedelic experiences, the enigma has been solved through the theory of set and setting parameters that predict and explain how the set and setting surrounding the experience have the power to influence and design the opposing psychedelic experiences (17, 20, 25–29). However, this phenomenon is still an enigma concerning childbirth [see also (10)].

**FIGURE 1:**
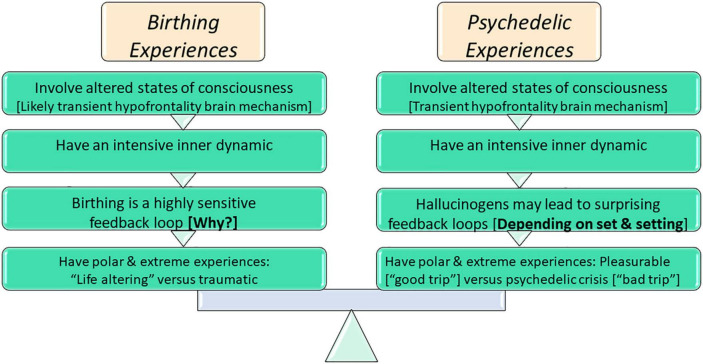
Comparing birthing experiences to psychedelic experiences.

Because of the similarities between birth and psychedelic experiences (illustrated in Figure 1), I argue that it would be useful to explore whether set and setting criteria would also help to solve the enigma in the case of childbirth experiences. I also aim to show that analyzing the opposing extreme ends of birth experiences in terms of set and settings would offer empirical support for the hypothesis that almost all set and setting parameters can be controlled. Thus, with the use of set and setting parameters, childbirth could be navigated to be more physiological and more often a positive experience.

In section “2. Birthing consciousness: The unique altered state of consciousness during physiological birth,” I elaborate on “birthing consciousness,” which is the altered state of consciousness that many women go through during physiological childbirth, and on the extreme polar ends of the birth experience. In section “3. Set and setting as an explanation for the extreme ends of altered states of consciousness experiences,” I explain set and setting parameters and how they diminish the enigma of opposing and extreme psychedelic experiences. In section “4. The new hypothesis: The same set and setting key parameters shape both psychedelic and birthing experiences,” I offer an analysis of the human birth physiology and experience in terms of set and setting and show how the theory can also solve the enigma concerning opposing birth experiences. In section “5. Discussion: Designing and navigating the childbirth experience rather than controlling it through medicalization,” I discuss the difference between “controlling” birth with techno-medical methods and “navigating” birth using set and setting framing, and I suggest a model for empirical research. Section “6. Conclusion: How the science of consciousness can promote the science of birth” is a summary of the paper and its claim that the same set and setting key parameters can help design, shape, and thus also navigate both psychedelic experiences and birthing experiences, concluding with the suggestion that the science of consciousness in general, and psychedelic research in particular, can promote the science of human birth.

## 2. Birthing consciousness: The unique altered state of consciousness during physiological birth

Birthing consciousness is an extremely positive altered state that women can experience during physiological childbirth (9, 12, 30). Labor contractions usually start easy, and intensify as labor continue (31). Women who experienced physiological natural childbirth describe a “transcendent” experience (32), with sensations of being in another zone, or another planet (11, 33), which is a less communicative state (13). It appears as a healthful dissociative state (33–35), because as physiological labor continues, although the pain of contractions intensifies, women report being calmer, along with reduced pain perception (36, 37).

I have suggested (12) that this specific altered state of consciousness during birth shares the same brain mechanism as other altered states of consciousness that have similar phenomenological and cognitive features. This brain state is the transient hypofrontality brain mechanism: the downregulation of prefrontal cortex function. I have hypothesized (12, 30) that transient hypofrontality is a key to natural birth, because this specific brain state helps a woman to cope with labor stress and labor pain [see also (9)].

### 2.1. The polar extremes of childbirth experiences

The birthing experience vastly effect the birthing person, not only in the immediate PP (35), and the method of birth is a significant factor influencing the birthing experience (11, 34, 35, 38). Commonly, the delivery methods used in childbirth are categorized as being one of two types: physiological birth (natural birth) and birth with medical intervention (such as Epidural anesthesia, Pitocin, instrumental delivery, episiotomy, or cesarean birth). However, it is more accurate to view delivery methods as falling on a spectrum—ranging from a birth with no interventions or professional assistance at all (what is called freebirth or unassisted birth) to the most medicalized birth. Having this spectrum perspective allows us to take into account that most Western birthing persons today choose a standard medically managed hospital childbirth, and even the minority of women who choose homebirth choose to be assisted by a childbirth professional (39). Hence, discussing physiological birth as if it were a completely natural birth with no medical intervention or professional assistance is not accurate. Thus, in referring to physiological or natural birth here, I am referring to childbirth that is less medicated and less disturbed, even though the birth may be occurring in a hospital with some minor medical intervention.

Around one-third of women describe their birthing experience as a traumatic, and nearly 85% of women experience varying degrees of mood disorders after childbirth (8). However, other women after birth report they have had an extremely positive birthing (40), and refer to feelings of euphoria, amazement, and awe, particularly after a physiological birth (11, 32, 33, 41). Natural birth is often described by women as a life-changing experience conferring a sense of inspiration, achievement, and empowerment (42, 43). Women have reported intense feelings of achievement, joy, and pride immediately after natural birth (33). These sensations, combined with the endorphins released during natural birth, lead to the PP phenomenon known as the “superwoman syndrome”: immediately after giving birth naturally, the birthing woman feels as if she can accomplish anything (41).

Concerning maternal mental health, it was found (8) that a highly medicated birth (instrumental births and emergency cesareans) is linked to mental disorder symptoms— such as somatization, depression, and anxiety [see also (11)].

Olza et al. (14), p. 11) stresses the importance of understanding the altered state of consciousness in physiological childbirth:

This description of women’s experiences during labor and birth and its potential for transformation resembles descriptions of mystical states of consciousness. Classically these states have been achieved through meditation and religious practices (including dancing, praying, and fasting) or through intake of substances with hallucinogenic properties such as psilocybin or LSD, which interact with serotonin receptors. Childbirth has not been mentioned in those classical descriptions. *The experience of spontaneous altered states of consciousness may well be a hallmark of physiological childbirth in humans and therefore its research may offer a unique opportunity to understand consciousness and transcendental growth* [emphasis added] … This knowledge is important to include in birth preparation courses and consultations.

Thus, I maintain that the existence of polar extremes of the birth experience, with opposite sensations and consequences, calls for investigation using consciousness studies tools.^2^

## 3. Set and setting as an explanation for the extreme ends of altered states of consciousness experiences

Since Leary et al. (44) coined the concept “set and setting,” it has become a fundamental concept in psychedelic research. The theory of set and setting argues that psychedelic experiences are shaped, first and foremost, by the mindset of an individual entering a psychedelic experience (set) and by the environment in which the event occurs (setting) (20, 45). The current accepted view is that the psychedelic experience depends on the set and setting.^3^ The set includes the personality, preparations, expectations, and intentions of the person having the experience, and the setting includes not only the physical location but also the people around and the broader sociocultural context (social setting, cultural setting, and relationship with other people) and the important elements of the freedom to exercise autonomy and access to psychological and physical support (20, 26).

Research began in the 1950s, when the Western world began to discover psychedelic substances. For nearly two decades, scientists studied their effects and during the 1970s, many clinical articles were published. However, studies dealing with the same substance produced contradictory conclusions [(20), pp. 67–93]. In fact, there were two polar perspectives concerning psychedelic substances at that time—the psychedelic perspective and the psychomimetic perspective—which affected the design, results, and interpretations of experiments (27). While the psychomimetic studies concluded, for example, that LSD creates a psychosis-like state, the psychedelic studies concluded that LSD could heal one’s mind. While the psychomimetic studies concluded that LSD induces anxiety, impairs cognition, and causes disturbances in perception, the psychedelic studies maintained that it induces euphoria, enhances cognitive abilities, and sharpens perception. While the psychomimetic studies concluded that LSD creates a traumatic experience, the psychedelic studies concluded that it creates a life-altering experience [(20), pp. 23–50, 67–93].

Scientists from each perspective presented research results that supported their claims, so the question arose as to how these claims could be so different. The answer turned out to be set and setting. The effect of psychedelic substances is not uniform, but depends on the individual and the situation (26). In other words, non-pharmacological variables have a vital part in the effects of psychedelic substances (46), thus enabling us to predict, to some extent, individual responses to psychedelic substances and help maximize potential benefits and reduce risks (25). It turned out that there were negative set and setting conditions (rigidity, unfamiliarity, non-acceptance) and positive set and setting conditions (flexibility, familiarity, acceptance). With the proper set and setting conditions, people reported positive and useful altered states of consciousness experiences and even that their lives had been changed for the better (20, 47).

**FIGURE 2:**
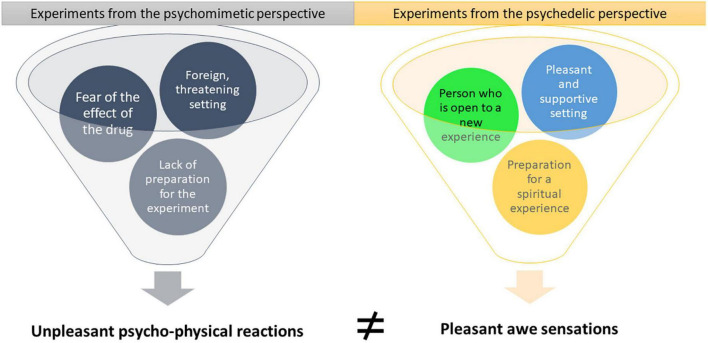
Different perspectives lead to different set and setting, thus contradictory results.

Figure 2 sets out the essential differences between the set and setting of the experiments performed during the 1960s and 1970s, depending on the perspective of the researcher. In the psychedelic experiments, the subjects expected a new and exciting experience, and enjoyed a pleasant and supportive environment. The researchers explained to the subjects that the psychedelic experience could sometimes be frightening and very intense but promised them one-on-one support and instructions on how to deal with the difficulties. In contrast, in the psychomimetic experiments, the subjects were not prepared and did not have support during the experiment. Thus, it is no wonder that their experiences were quite opposite and that the conclusions concerning the effects of the psychedelic substances contradicted one another [for a detailed review, see (27)].

## 4. The new hypothesis: The same set and setting key parameters shape both psychedelic and birthing experiences

As was discussed in the section “1. Introduction,” birthing and psychedelic experiences have similarities that can be explained by the set and setting theory. And, as shown in the previous section, the framing of set and setting also has the power to predict future outcomes of such experiences.

The basics of the set and setting theory contradict one of the most fundamental and rooted premises of modern obstetrics—that birth operates as a purely physiological mechanism. In other words, there are body parts, mostly the womb and the pelvis, that function to achieve the goal of ejecting the fetus out of the birthing body. But the birthing woman also births with her mind, not just with her body (9, 34). Thus, her consciousness and her experiences also function during birth, and the physical and social setting has a crucial effect on these body parts. As long as modern obstetrics maintains its flawed assumption regarding childbirth, it will have no real opportunity to seriously acknowledge crucial physical, mental, and social effects on the process of birth (i.e., the set and setting parameters), in spite of increasing bodies of research that empirically validate these kinds of effects. In the following subsections I provide examples of such empirical studies.

### 4.1. Preparation (set)

In psychedelic research, the term “preparation” refers to the psychological, and perhaps even physiological and environmental, preparation of the person who is about to have the altered state of consciousness experience [(20), pp. 95–128]. This preparation is, of course, also important in the birthing experience. However, there are psycho-physiological, automatic preparations for birth, such as biochemical (31) and brain changes (48). I recently offered a new hypothesis concerning psycho-physiological *automatic preparations* for birth (10). Various researches concerning the maternal brain during pregnancy and after it, reveal remarkable neuroplasticity, and functional and anatomical changes (49–51). While the generally accepted perspective is that these brain changes tend *to prepare the maternal brain for motherhood*, I proposed a complementary perspective, arguing that some of these changes before birth can be *preparations of the maternal brain for childbirth* (48). It seems plausible that the birthing brain is hard wired to experience the unique altered state of consciousness during physiological birth, i.e., birthing consciousness. It seems that the brain is preparing itself for the birthing process, that physiology supports phenomenological characteristics of a focused and calm state (10, 12).

There are also hormonal preparations and activation during birth itself that ameliorate the physiological birth process. Various hormones initiate and maintain the process of birth: oxytocin, endorphins, prolactin, beta-endorphins, and dopamine (31). These biochemistry mechanisms also affect the feelings and reactions of a birthing person (9, 52). For example, beta-endorphins increase pain tolerance (31, 53). The alteration of the pain perception during natural childbirth empowers the possibility for the woman to experience birthing consciousness, with all its psychological and physiological benefits (9).

Thus, it seems that unlike the intentional personal preparation for a psychedelic experience, in the case of preparation for childbirth’s special altered state of consciousness, the body and brain of the pregnant woman automatically, adaptively, has 9 months to prepare itself for the birthing hurdle and experience. Of course, there are various social and cultural disturbances to this process, which will be discussed in the following subsections.

### 4.2. Intentions and expectations (set)

Studies indicate that there are strong connections between the beliefs and perceptions of the birthing woman before the event of birth concerning the birth process and experiences and birth outcomes, in terms of the birth method and the subjective birth experience (54, 55). The practical conclusion of these studies is that enhancing the belief of women, before childbirth, that they can have a physiological birth and that they have the necessary psychological ability (can handle the childbirth pain) and physiological capacity (their body is capable of birth) can decrease fear of childbirth and also reduce the increasing rate of medical obstetric interventions. In another study interviewing midwives concerning their methods of protecting birthing women’s perineum during the second stage, the emerging theme was that the fear of the birthing woman correlated with perineal tears (56).

It was empirically validated that the birthing women’s beliefs concerning birth–natural process vs. medical procedure–are linked to preferences concerning the birth place–a more natural birthing place and a physiological childbirth vs. a typical hospital birth managed from a medical point of view (57). Interestingly, the beliefs of birthing women regarding birth predicted their preferences better than they predicted their actual birth experience. Research indeed indicates that birth beliefs are crucial in the initial decision-making process concerning birth options. However, as the authors of this study demonstrated, in the current medicalized obstetric arena, women who want natural childbirth usually must vigorously insist on it (57).

This arena may unintentionally sabotage women’s beliefs and plans for more natural childbirth. It also has psychological effects such as anxiety, stress, and nervousness, which raise the probability of dysfunctional childbirth (54, 58). Empirical research shows that elevated levels of epinephrine are caused by fear and anxiety, and during childbirth are linked to less powerful uterine contractions (31, 59). It is probably because fear or anxiety affects the oxytocin system, which has a significant function in promoting contractions (60). Thus, in times of stress or fear, labor does not progress (37, 61), hence leading to more medical interventional childbirth (9, 54, 62).

The feelings of fear, stress, and anxiety have adverse effects also from a biochemical point of view concerning pain, because these sensations hinder anti-nociception (63). The sensation of labor pain itself is not necessarily evaluated negatively by women during physiological birth. For example, Whitburn et al. (64) show that women describe their pain experiences as shifting between two mindsets during the physiological childbirth: a mind that is determined to accept the pain, vs. a mindset of suffering and rejecting the pain.

It is interesting that the intensity of labor pain is not linked to a negative birth experience (9, 64). On the contrary, during physiological childbirth, when no epidural anesthesia is performed, women sometimes experience joy and thrills, not misery (63). These feelings can be comparable to the pain that thrill seekers are facing during an adventure (30, 65). For example, women who delivered in a planned home births described labor pain positively and referred to their desire to accept and master labor pain, signifying they manage their own health and wellbeing (66). A birthing woman may experience greater pain than she has ever experienced before, but it depends on her mindset as to whether she refers to this pain as suffering. Many childbirth educators conclude that when a birthing woman understands the physiological explanation for childbirth pain, she can view the escalating pain as encouraging because it indicated that the birth is progressing (9, 40, 62). Thus, it is not a negligible phenomenon that many birthing women experience the intense labor pain together with feelings of triumph, inspiration, and pride. In these situations, the birth experience can improve wellbeing (11, 32, 33, 35, 41, 63).

For a birthing person who experienced birthing consciousness, withdrawing, and accepting the positive function of labor pain was beneficial. Submitting to the painful natural birth process was motivated by inner power and purposeful decision. In words from set and setting theory, it stemmed from knowledge, self-preparation, and self-intention toward birth.

### 4.3. Physical environment (setting)

Many recent studies focus on the physical setting of the birth space and how it may impact the process of childbirth and method of delivery. In other words, the physical childbirth environment can support physiological birth or hinder it (67–70). In relation to birthing consciousness, there are aesthetic and physical aspects that tend to activate neocortex regions, such as loud voices, high irradiance lights, and also the knowledge you are constantly being observed by strangers (9, 12, 13). Activity in these areas of the brain is in tension with hypofrontality brain mechanism. Therefore, such conditions prevent the woman to access the state of birthing consciousness (9). Yet, the proper birth arena may encourage birthing consciousness. One example are low irradiance lights in the birth room, that were found to be linked to less emergency medical interventions (70). Another example is removing the standard birth bed from the center of the birth room (68, 69). This aesthetic change explains that horizontal position during birth is linked to more prolonged birth and complications that necessitate emergency medical interventions. Reclining during delivery is also considered more painful, thus frequently leading to epidural anesthesia, which raises the risks of hyper-medicalized childbirth (71).

The birth arena also shapes the behavior of the birthing woman. Women laboring in hospitals tend to behave more passively. But in more homey childbirth setting, such as natural birthing rooms in hospitals or birth centers, women instinctively claim ownership of their surroundings. They tend to behave more actively, for example by changing body positions during birth (72), and it is well known that changing positions during labor promotes physiological birth (73).

The issue of design and aesthetics of the birth space might also be related to the psychological need to feel safe, which, as noted by many [see (13, 36)], is a significant factor in promoting a physiological birth and ensuring psychological comfort. Women describe their deep urgency and desire to be in a sheltered place as contractions intensify and become more painful and how the feeling of safety helps them focus on each contraction. This inherent need is described by birthing women from a social support perspective and an environmental space perspective (74, 75). This need to feel safe and secure to promote the birth process and its connection to physical aspects of the birthing environment was uncovered decades ago in rodent studies. Newton et al. (76, 77) were the first to experimentally demonstrate the environmental regulation of parturition in laboratory mice. Proceeding a series of studies in laboring mice, it was concluded that the labor of mice functions best in a sheltered and undisturbed atmosphere, such as hidden container, as opposed to a glass container. More recently, environmental disturbance during labor in dogs and cows was found to extend parturition (59, 78). In spite of these findings, the typical modern hospital setting includes unfamiliar sounds, voices of strangers, and strong lights and smells. All these aspects are related to catecholamines release in the birthing woman, which can cause neocortical activity and disturb the process of birth [(9, 53), pp. 30–39].

It should be remembered, though, that designing better birthing rooms cannot be counted as a magical solution for supporting natural births; they are merely one factor in the setting and not necessarily the determinative one. The set of the preparations and intentions, e.g., the will, of the women play a crucial role in the outcomes. Another crucial role plays the hospital’s birth philosophy, a function of setting. A recent study (79) emphasizes the crucial role of the birth philosophy of medical staff, which reflects the hospital authorities’ perspective toward childbirth in creating or denying a birth setting promoting a positive birth experience. Two different childbirth spaces were used. One space was designed from a medicalized birth perspective, where a woman is viewed as a passive agent, and birth is considered a dangerous medical event. The second space was designed from a more physiological birth perspective, where a woman is viewed as an active agent, and childbirth is viewed as a natural process. Interestingly, the different birth spaces’ different designs did not help improve birth outcomes because, according to the researchers, the hospital’s medicalization approach also invaded the second space, thus influencing the ambiance and the attitude toward the birthing women.

### 4.4. Support from and relationship with medical professionals (setting)

One-on-one support decreases the rate and need of various medical interventions (80) and increase the satisfaction of the birthing woman concerning her birth experience (81). Still, while continued support during birth is crucial, and some argue that it should be considered a basic human right (82), in many of typical birthing rooms, hospital systems and procedures rarely prioritize, or support, birthing women’s sense of agency and choices (57). As with findings presented in the previous subsection, there is a link between discounting the innate need for care and support during the birth process, with rising rates of hyper-medicalized childbirths (83–85).

Even the historical meanings of the words “obstetrician” and “midwife” reflect the importance of their relationship with the birthing women. *Obstetrician* comes from the Latin phrase *obstetrics*, which plainly means “she who stands before,” which refers to “midwife.” Moreover, *midwife* is an old German term that simply means “with woman” (86). Many studies have demonstrated that birth outcomes are improved for birthing women who have continuous support during childbirth from a doula or a private midwife, who in both cases use non-pharmacological pain management strategies. These methods promote physiological birth, with less perceived pain, and more positive subjective experiences (87, 88). In fact, there is a well-known saying that “if a doula were a drug, it would be unethical not to use it” (87). However, in the power relationship dynamics in and around the typical birthing room in a Western hospital, it seems there are at least three kinds of power imbalances that most often do not favor the birthing woman (an issue that is crucial to the concept of setting). Dahan and Cohen Shabot (34) show that not only are there power imbalances between the birthing women and the staff in the delivery room, there are also power imbalances within the staff (i.e., doctors vs. midwives) and between the staff and hospital management. For example, in many obstetric systems the tendency is to manage childbirth process from the perspective of avoiding risks for the hospital (such as expensive lawsuits), rather than for the birthing woman. Thus, the hospital management imposed routine practices that tended to underrate the importance of women-centered approach that acknowledge the importance of women’s subjective birthing experience (79). This finding is crucial, because the hyper-medicalization approach in most of Western hospitals today appears to conflict, rather than support, many birthing person’s sense of agency (57).

Kitzinger (89), a social anthropologist, also described a general negative reduced sense of agency of women throughout childbirth. In most hospitals birthing women are forced to wear a hospital gown. But giving up a person’s own wearing sometimes means giving up a person’s individuality and choice. Further, in several ways, the birthing woman is supposed to act as a passive patient—to follow instructions, not interfere with the medical staff, and be a “good girl” as if she were a child and not a grown woman (89). Indeed, many women refer to being disempowered during birth in typical modern hospitals (39). Notably, women from developed countries regularly portray their struggle during birth to avoid unnecessary interventions. These birthing persons are often powerless in their effort to refuse medicalized birth, usually because of the hyper-medicalization approach, that is common in many western hospitals, which is translated to medial protocols (9, 39, 90). This finding appears to illustrate the complex relationship between the birthing woman and the medical staff; although they are supposed to support her, they frequently do the opposite. Participants in studies stated:

“I…thought if the midwives and doctor left me alone I could most certainly birth my baby.” [(39), p. 99].

“I was steamrolled with unnecessary intervention and did not get to speak with a doctor about my options, risks vs. benefits… I feel like the nurses, doctors and hospital only did what was in their best interest, not mine… It was a nightmare.” [(90), p. 4].

Reed et al. (90) did an online survey of 748 women, asking what they found to be the most traumatizing during childbirth. The major issues reported were the actions of medical staff and their interaction with them. Women felt that the medical staff prioritized their own agendas on top of the wishes and needs of the birthing woman. Many examples reveal the sometimes-aggressive attempts of the medical staff to convince the birthing woman to agree to unnecessary intervention. Women also described actions that were violent and abusive (90). These themes are all parts of the phenomenon called “obstetric violence”—the ill-mannered and insulting treatment, verbal or physical, of birthing person during the event of birth, which is acknowledged as a worldwide problem [see (91–93)]. For example, medical staff sometimes use threats and twisted-partial facts, usually related to the wellbeing of the fetus, to coerce the birthing person into complying with procedures (90, 94–96). For some women, the actions of the medical staff during the event of the birth triggered memories of sexual assault (90).^4^

A recent review (97) of what is currently known about birth trauma confirmed that it is not directly related to medical pre-existing factors. Three key themes regarding birth trauma, highly relevant to the setting issues here, were identified: support during birth, the birthing person’s feeling of knowledge and control, and the quality of care provided by the professionals (97). The attitude of the personnel in the delivery room has a crucial effect on the birthing person from psychological perspective (98). Thus, there is a serious need for childbirth care providers to be trained as to how important a positive relationship with the birthing woman is in terms of physiological and psychological health (90).

### 4.5. Ability to exercise autonomy (setting)

Dixon et al. (36) notice that the intense absorption present during physiological childbirth reminds the mental state of “flow”: despite the dissociation (concerning time and space), along with the sensations of concentration and loss of self-consciousness, a sense of personal control emerges and retained (36). Negative birthing experiences are described as those that suppress birthing person’s sense of autonomy and control (99). These sensations and feelings usually emerge during emergency, highly medicalized labor. In these cases, women describe experiencing a negative dissociative state (100, 101). However, these negative experiences happen not only during emergency births. As discussed in previous sections, this can include being forced to wear patient gowns, being expected to act passively, and being unable to choose their birthing position. All these issues are linked to adverse emotions of losing choice, control, and autonomy (89).

In particular, the freedom to choose one’s birthing position contributes not only to the psychological feeling of control and autonomy but also promotes physiological birth. Reitter et al. (102) were the first to assess dimensions of the pelvis of women (pregnant vs. non-pregnant) in several positions, using MRI. They found that a kneeling squat position strikingly and significantly increases pelvic dimensions: “increase in the transverse diameters of the mid pelvis and the pelvic outlet (0.9e1.9 cm) when women change from the supine dorsal position to a kneeling squat position” (p. 662, e7). They also found that this increase in pelvic dimensions is even more prominent in pregnant women. This means there is great potential in changing positions during birth for easier delivery, because increased pelvic diameters provide an anatomic boost for simpler descent of the fetus during childbirth. Unfortunately, Reed et al. (90) show that in many cases, even birthing women without epidurals are forbidden to move about freely and instead are forced to lie down.

### 4.6. Cultural factors (setting)

It has been found that prevalent descriptions of childbirth in popular media (e.g., television and film dramas and reality television) perpetuate the medical approach to childbirth, while coverage of more physiological births is generally absent from the media (103). In a sense, the common and accepted image of childbirth in Western culture is a sterile one: from the moment of epidural anesthesia, the birthing woman lies, relaxed, in the delivery room, connected to monitoring devices, free from labor pain. Labor pain is something to fear, something archaic—a thing from before the age of reason and high-tech medicine. Labor pain is introduced in popular mass media as a necessary evil that needs to be gotten rid of, as soon as possible, in contemporary labor in a hospital. After anesthesia is administered, the birthing women is, in a sense, rescued from her own body, thus, she is detached from her body (104). However, although her fear and pain is terminated, so is her own autonomy (105). Moreover, birthing women’s bodies are presented in reality shows as incapable of physiological birthing, thus requiring technological help, medical interventions, and surveillance throughout the birth process. These shows represent the birthing women’s body as inferior (106). These depictions have real-life effects. For example, students from the University of British Columbia, young adults who had been socialized into a medicalized birth culture, were found to fear vaginal childbirth and prefer an epidural or elective C-section over a physiological birth (107).

This picture of childbirth is inaccurate, however, it does not show what usually happens in the advanced stages of medical birth, such as the association between epidural anesthesia and instrumental and emergency cesarean births (108); the correlation of medical births with a precarious mental state after birth, such as PP depression and post-trauma (8); and potentially more challenging PP physiological recovery, for example in terms of pelvic floor health in cases of buffer incisions (109).

Another example of the effect of cultural factors is the rising use of epidural anesthesia in Western countries. While epidural is highly effective form of pain relief, it does not automatically improve the birthing woman’s experience (34). Social support during labor was found to be much more crucial factor than epidural in improving birth experience, although underestimated (110). Birthing women without epidural anesthesia had shorter births, with more chances to have a more natural birth and less interventional birth (111). This is not to suggest that epidurals always have negative effects, but although technological innovations have significantly reduced mortality of birthing women and babies, many technological interventions have become needlessly routine (9, 71). These technological interventions, such as epidurals and electronic fetal monitoring (EFM), convert a typical low-risk birth from a physiologic process into a medical process.

Electronic fetal monitoring is usually used in admission to the hospital with no consideration of the risks in using it continuously during the birth process (9). A continuous use of EFM in low-risk birthing women sometimes starts a chronological sequence of interventions that increase the risk for an unplanned C-section (71, 112). Here is typical example of a cascade of obstetric interventions (113): lying for monitoring sometimes weaken contractions, which require synthetic hormonal induction (Pitocin), which necessitates that the monitoring and lying continues. Pitocin, and lying on the back with no ability to move, lead to intense pain experience. This negative pain experience increases the need for a pain reliever, such as epidural. Unfortunately, epidurals are linked to lengthier births and unproductive pushing, thus increase the risk to instrumental births, such as forceps or vacuum extraction. Instrumental births are linked to perineal cuts (episiotomy). If an instrumental vaginal birth fails, an emergency C-section is necessary to rescue the woman and her child (61, 71, 112, 114, 115). Empirical studies confirms that even a minor intervention increases the risk that a birth will end up being highly medicalized (9, 108).

Another example of the effect of cultural factors are studies that show that ethnocultural differences, such as language, values, or religious beliefs–can affect women’s perceptions and beliefs about childbirth, and also influence birth experiences, even when there is no difference in the levels of medical interventions (116).

### 4.7. The set and setting theory concerning childbirth

The analysis I have offered shows how the same set and setting key parameters that shape psychedelic experiences also shape birthing experiences. And, as stated previously, it is not only that the set and setting theory has explanatory powers to describe the birthing experience retrospectively. The framing of set and setting also has the power to predict future experience outcomes.

**FIGURE 3:**
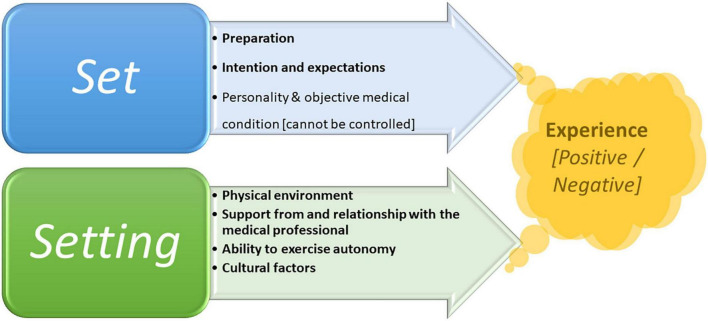
Set and setting parameters that shape both psychedelic and birth experiences.

Figure 3 shows the set and setting key parameters that enable us to shape and navigate the birth experience, and even birth outcomes, in most cases. It also shows which objective factors we cannot control, i.e., personality, life history, and objective medical condition. Indeed, many personal factors can affect the birth experience and consequences, such as the life history of the birthing woman, previous trauma, current social and psychological state, and her physiological condition and obstetrical status [see, for example (117)].

## 5. Discussion: Designing and navigating the childbirth experience rather than controlling it through medicalization

There seems to be broad agreement with the familiar slogan that “you cannot control birth” (118). Perhaps this is because physiological birth is broadly conceived as a dangerous process that can escalate in an instant toward becoming an emergency medical event. This perspective is usual in typical Western hospital environments (9, 12). Obstetrics has always viewed childbirth as a physical event, focusing on the uterus contractions, the opening of the pelvis, and the fetus’s wellbeing: how to finish the event with a healthy mother and child, while preventing many pathological escalations (119, 120). This attitude is not different today [see, for example (121)]. Even in studies of how to minimize PP-PTSD, one of the interventions offered is to instruct women to have realistic expectations concerning birth, to be open-minded about the birth process, to accept that it is unpredictable, and, for example, to exchange their “birth plans” for “birth flow charts” [see for instance (122)]. Women are encouraged to accept in advance that they will not be able to control or manage their birth process and, in general, should hand over power and control to others (i.e., to obstetric and other health care professionals).

In the late 1980s, Newton (123) discussed many social and environmental effects that might promote birth or hinder delivery, and offered that obstetrics should pay attention to and study them, in order to gently control the physiological birth process, navigating it to a safe and healthy place using simple social, psychological, and environmental tools:

Unfortunately, the psychologic aspects of labor regulation get sparse attention in academic texts. Every day in labor and delivery suites it is noted how labor slows in many women at the time their environment changes from home to hospital and from labor to delivery room. Much more well-controlled research is needed on the environmental regulation of labor. It may be especially important to know which environmental factors inhibit or promote normal human labor. Randomized controlled trials of many aspects of current obstetric procedures that have a psychologic and environmental impact on the laboring woman are especially needed. *I hope that the decade of the 1990s will see this knowledge developed and used to help childbearing women* [emphasis added]. [(123), p. 108].

Unfortunately, Newton’s hope was not fulfilled. There is a continually rising rate of medical intervention during childbirth (124), instead of navigating the childbirth process in the environmental and social ways suggested by Newton and confirmed by many others since then. Keeping in mind that studies specify that the increasing C-section childbirth rates in the United States, and the hyper-medicalization attitude, in general, do not contribute women’s physiological and psychological health (4, 9, 125), it seems that hyper-medicalization is not the right strategy if the goal is to manage childbirth for the sake of the mother’s and the infant’s wellbeing.

I believe the central tragedy here is the failure to acknowledge that birth is a complex psycho-physiological, social, biochemical process. Trying to control it by more medical equipment and procedures (such as routine EFM during labor, routine inductions, routine anesthesia), in order to be ready for any possible emergency, probably itself plays a significant role in causing pathological escalation. Many empirical studies on the cascade of interventions during birth demonstrate the path of disturbances to physiological birth: the various mental and environmental variables noted previously that impede childbirth from progressing [see (61, 64, 71, 113, 126, 127)].

It seems reasonable that the broad agreement that birth cannot be predicted or controlled without medicalization is related to the common basic assumption that delivery is dangerous and harrowing and that before the era of modern obstetrics, there was nothing that could be done about it from the perspective of the birthing woman herself, aside from being close to potential helpers around the time of birth [see (128)]. The belief that “you cannot control birth” has convinced birthing women to allow medical professionals to take over, because medical professionals believe that the only way to increase control and safety during childbirth is through hyper-medicalization. However, I propose a better way is to help women design and navigate the childbirth experience, using set and setting theory. One can, in most cases, *navigate* birth to a physiological direction if one does not ignore the birthing woman’s mental state and the environmental and social settings.

The experience of childbirth does not uniformly emerge in all women in the same way or even in the same women in different births. Far from uniformity, the effects of childbirth are remarkably diverse and sometimes polarized. I suggest that the birth experience depends first and foremost on the set (the psychological variables of personality, preparations, intentions, and expectations of the birthing woman) and the setting (the variables that include the physical, social, and cultural environment in which childbirth occurs).

Thus, for example, the birth experience that takes place in a threatening environment for an anxious woman who is afraid of the pain of birth is likely to provoke unpleasant reactions, anxiety, intense pain, and suffering, to the point of complicating the birth and causing post-traumatic effects. On the other hand, a woman who is aware of the issue of pain management in childbirth and seeks to maximize the therapeutic and analgesic effects of the hormones that are part of the birth process, with childbirth taking place in a supportive and pleasant environment, could be expected to have a more positive, even empowering, experience.

**FIGURE 4:**
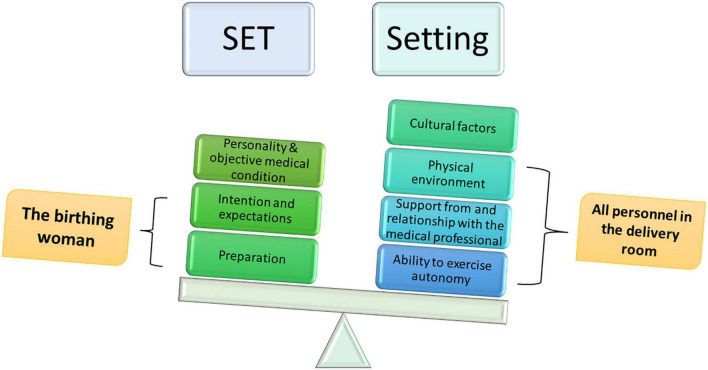
How can the science of consciousness promote the science of birth?

My hypothesis is that differences in set and setting conditions are what lead to opposite extreme results in the birth experiences of many women, just like differences in set and setting conditions in psychedelic research in the mid-20th century revealed traumatic experiences, on the one hand, and positive and life-changing experiences, on the other. In other words, I suggest a conceptual shift. Instead of thinking in terms of “control” regarding childbirth, it would be better to think in terms of “design and navigation.” Instead of trying to control childbirth by hyper-medicalization of it, we should design the birth set and setting to provide optimal conditions for the birth experience and thereby navigate birth. Figure 4 illustrates the set and setting theory in relation to the birth arena.

### 5.1. The set and setting model concerning physiological childbirth is ripe for empirical investigation

The history of psychedelic research directs us to give more attention to how non-physiological factors shape altered states of consciousness and their effects on the mind and body. During childbirth, the woman responds *immediately* to the set and setting key parameters, because birthing has intensive feedback loop dynamics. Thus, concerning optimal management of the birth process, set and setting are crucial factors we must deal with. If my hypothesis is valid, then the science of consciousness and philosophy of mind can promote the science of birth.

**FIGURE 5:**
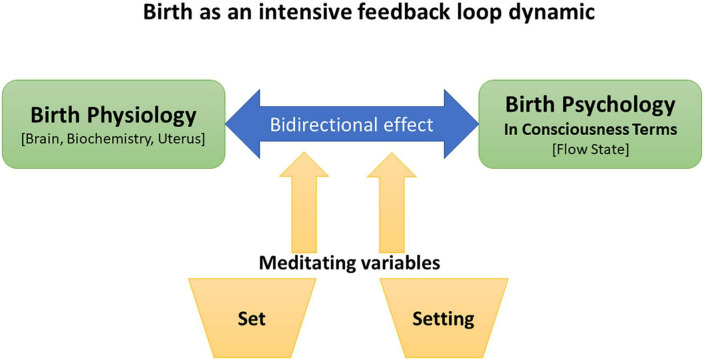
The model of physiological birth in terms of set and setting.

Figure 5 illustrates the hypothesis I offer, concerning a unified model of the psycho-physical event of physiological human birth. This complex dynamic shows the ability of many factors to *enhance* the positive state of consciousness or *suppress* it. The result is an extreme experience: a positive and life-changing experience compared to a negative or even traumatic one. I believe that this model is ripe for empirical inquiry.

## 6. Conclusion: How the science of consciousness can promote the science of birth

The seeming unpredictability and uncontrollability of the birth process and the possibility for unexpected complications should not encourage fatalism. The set and setting concept may significantly advance scientific understanding of the birth process, particularly what promotes healthy birth and what may hinder it. The history of psychedelics’ extreme polar experiences directs us to give more attention as to how non-physiological factors shape altered states of consciousness and their effects on the mind and body. In this paper, I have theoretically demonstrated that a broader understanding of the set and setting key parameters can significantly improve our understanding of human birth.

Moreover, the basics of the set and setting theory challenge one of the fundamental and rooted premises of modern obstetrics: that birth progresses as it does purely from a physiological mechanism. But, as stated, women also give birth with their minds, not just their bodies. Thus, physical and social settings have crucial effects on the birth process, and there are empirical studies that validate these kinds of effects. In examining birth experiences, we must re-examine the implications of the altered states of consciousness in childbirth, which respond immediately and flexibly to set and setting key parameters. For optimal management of the birth process, set and setting are crucial factors.

## Data availability statement

The original contributions presented in this study are included in this article/supplementary material, further inquiries can be directed to the corresponding author.

## Author contributions

The author confirms being the sole contributor of this work and has approved it for publication.

## Footnotes

1 It is acknowledged that not all birthing persons are *cis* gender women. The terms “women” and “mothers” and female pronouns are used throughout this paper for the sake of simplicity, but the analysis applies to the birthing experiences of all birthing persons.

2 I acknowledge that there are women on the spectrum between the extreme ends of the birth experience who experience fewer intense sensations during childbirth and less significant consequences; however, these women are not the focus of the current paper. Future empirical research can determine the percentages of women with these different experiences.

3 Researchers have demonstrated that set and setting also play a crucial role in shaping the effects of diverse psychoactive drugs such as alcohol, methylphenidate (Ritalin), methamphetamine, cocaine, crack, and heroin.

4 The phenomenon of obstetric violence, although highly relevant to this subject, is beyond the scope of this paper.
